# Lights and Shadows on Artificial Intelligence in Glaucoma: Transforming Screening, Monitoring, and Prognosis

**DOI:** 10.3390/jcm14072139

**Published:** 2025-03-21

**Authors:** Alessio Martucci, Gabriele Gallo Afflitto, Giulio Pocobelli, Francesco Aiello, Raffaele Mancino, Carlo Nucci

**Affiliations:** Ophthalmology Unit, Department of Experimental Medicine, University of Rome “Tor Vergata”, 00133 Rome, Italy

**Keywords:** glaucoma, artificial intelligence, deep learning, machine learning, optical coherence tomography, visual field test, fundus imaging

## Abstract

**Background/Objectives**: Artificial intelligence (AI) is increasingly being integrated into medicine, including ophthalmology, owing to its strong capabilities in image recognition. **Methods**: This review focuses on the most recent key applications of AI in the diagnosis and management of, as well as research on, glaucoma by performing a systematic review of the latest papers in the literature. **Results**: In glaucoma, AI can help analyze large amounts of data from diagnostic tools, such as fundus images, optical coherence tomography scans, and visual field tests. **Conclusions**: AI technologies can enhance the accuracy of glaucoma diagnoses and could provide significant economic benefits by automating routine tasks, improving diagnostic accuracy, and enhancing access to care, especially in underserved areas. However, despite these promising results, challenges persist, including limited dataset size and diversity, class imbalance, the need to optimize models for early detection, and the integration of multimodal data into clinical practice. Currently, ophthalmologists are expected to continue playing a leading role in managing glaucomatous eyes and overseeing the development and validation of AI tools.

## 1. Introduction

The term glaucoma refers to a group of multifactorial progressive optic neuropathies characterized by optic nerve head (ONH) abnormalities, thinning of the retinal nerve fibre layer (RNFL), and characteristic visual field (VF) defects [1,2,3]. Glaucoma represents the leading cause of visual impairment and irreversible blindness globally [3,4,5].

The diagnosis of glaucoma relies on identifying characteristic structural abnormalities, which can be detected using ophthalmoscopy, colour fundus photography (CFP), or optical coherence tomography (OCT). Psychophysical functional tests—such as static automatic perimetry (SAP)—remain the gold standard for diagnosing glaucoma [6]. Several large, randomized controlled trials have demonstrated that discrepancies between structural and functional measures may occur. While many patients with glaucoma show structural changes before functional deficits become evident, functional abnormalities may occur before structural changes are noticeable [7,8]. In cases of moderate to advanced glaucoma, the diagnosis is usually straightforward. However, a major challenge is determining the best method to detect early glaucoma.

Artificial intelligence (AI) is an “umbrella term” that identifies software-based algorithms used to complete a given task [9,10,11]. AI relies on algorithms and statistical models to analyze data and identify patterns [11]. AI can be classified into three primary types: General Artificial Intelligence (GAI), which is a theoretical form of AI capable of performing all cognitive tasks that human intelligence can achieve; Narrow Artificial Intelligence (NAI), designed to perform specific tasks similar to human intelligence but within a limited domain; and Artificial Superintelligence (ASI), a hypothetical level of AI that surpasses human intelligence in all cognitive and functional aspects [12].

AI is rapidly advancing across various medical specialties, employing diverse technologies, applications, and algorithms to support disease prevention, diagnosis, and treatment. Two key AI-driven approaches are Natural Language Processing (NLP), which extracts and processes information from unstructured data sources, such as medical records and research articles, and machine learning (ML), which analyses structured data, including genetic profiles and medical imaging, to enhance secondary screening and diagnostic accuracy. ML enables computers to identify patterns and generate predictive insights by continuously learning from existing datasets [12].

Machine learning classifiers (MLCs), such as random forest (RF), logistic regression (LR), support vector machines (SVMs), Gaussian mixture models (GMMs), and independent component analysis (ICA), are commonly adopted ML clustering algorithms. AI techniques in ophthalmology can be categorized into two main approaches: deep learning (DL) and self-learning algorithms. DL, a term introduced in the 2000s, is one of the most modern and explored branches of ML. DL uses multiple neural network layers to improve prediction accuracy. In addition to classification tasks, DL automatically extracts relevant features for specific diagnostic purposes. These models are designed to recognize and classify medical images and are widely applied for retinal image segmentation and disease detection [12].

DL algorithms use representation learning techniques with multiple layers of abstraction to process input data, eliminating the need for manual feature engineering [13]. Projecting high-dimensional data onto a lower-dimensional manifold automatically recognizes complex structures within the data [13]. The multiple layers adopted by DL models resemble the network of connections found in the animal brain, and they are commonly referred to as artificial neural networks (ANNs). A specific subtype of ANN is the convolutional neural network (CNN), which specializes in image recognition and computer vision tasks. CNNs are particularly effective at processing high-dimensional data, such as fundus and OCT images, by extracting and analyzing features, such as texture, edges, intensity, and thickness. Through multiple layers of abstraction, CNNs identify important features related to specific tasks, such as detecting pathology or distinguishing tissue types [14,15,16].

Since ANNs have inherent limitations, structured data must be provided to train the model effectively.

Machine learning (ML) plays a crucial role in refining AI algorithms by optimizing predictive accuracy through iterative data processing [12].

**Figure 1:**
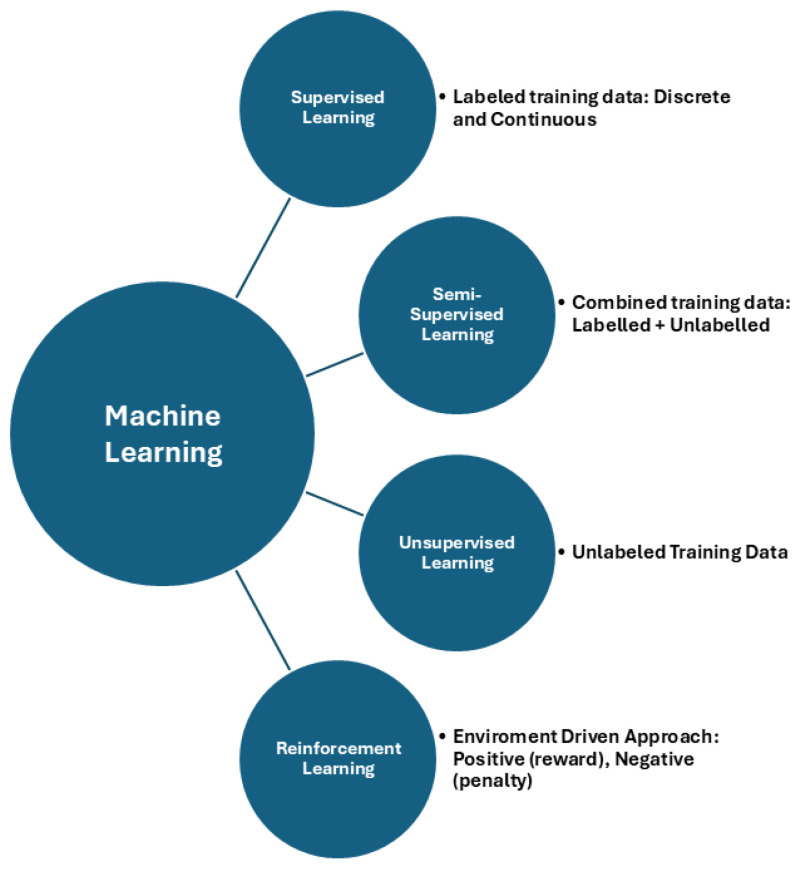
Machine learning branches.

To perform specific tasks, AI algorithms require training, which can be supervised, semi-supervised, or unsupervised depending on the degree of data labelling. Supervised models use fully labelled datasets, unsupervised models recognize patterns without labels, and semi-supervised models use a combination of labelled and unlabeled data. Reinforcement learning (RL) has emerged as a dynamic and transformative paradigm in AI, offering the promise of intelligent decision-making in complex and dynamic environments. This unique feature enables RL to address sequential decision-making problems using simultaneous sampling, evaluation, and feedback. After training, AI algorithms must be validated with a separate test set to avoid bias. Large datasets are required to ascertain the efficacy and reliability of AI algorithms, which is a limitation of this technology [14,17,18] (Figure 1).

AI has instigated profound metamorphosis in medicine. For example, in the field of radiology, AI redefined traditional workflows and elevated the radiologist’s role. In the realm of image acquisition, AI augments scanning procedures, optimizes image fidelity, and fosters sophisticated image reconstruction across magnetic resonance imaging (MRI), computed tomography (CT), and positron emission tomography (PET) modalities. Foremost, among these advancements, DL accelerates MRI scanning, harmonizing efficiency and quality, with commensurate progress witnessed in CT and PET image reconstruction [19]. To date, the FDA has approved 201 AI algorithms from 2008 to 2022. However, estimates are that the market for AI in medical imaging is going to grow by 10-fold over the next 10 years [20].

Demonstrating the potential of CNNs, the AI-driven segmentation of lung nodules from CT scans has achieved an impressive AUROC of 94.4%, surpassing the performance of six radiologists in early lung cancer detection and treatment. Likewise, CNNs have been instrumental in segmenting brain tumours from MRI scans and analyzing retinal images for early signs of diabetic retinopathy, highlighting AI’s extensive applicability and versatility in medical imaging [19].

Ophthalmology is also a key area for AI development, with digital tools such as VF testing, OCT, and retinal imaging enhancing diagnostic precision. AI applications in ophthalmology primarily focus on vision-threatening conditions, including age-related macular degeneration, diabetic retinopathy, glaucoma, and cataracts. The increasing prevalence of these conditions in the United States has driven the integration of AI-based diagnostic and management strategies.

Despite the success of AI in other areas of ophthalmology, such as diabetic retinopathy screening programmes, its clinical application in glaucoma management remains in the early stages of development [21].

A large body of evidence is currently available, suggesting that AI is a powerful tool whose implementation might aid in the interpretation of a wide array of clinical data, both functional (e.g., SAP) and structural (e.g., fundus photographs, OCT).

This paper reviews recent publications exploring the potential applications of AI technology in the management of glaucoma. Our goal is to analyze recent advancements in AI, as they relate to the tests commonly used for glaucoma diagnosis and follow-up, including structural tests such as OCT and fundus photography, as well as functional tests such as VF.

## 2. Clinical Applications of AI

The integration of computer science into clinical practice has shown promise for improving the accuracy of various processes. Among the different AI approaches, DL models have demonstrated high sensitivity and specificity in glaucoma screening using optic disc and excavation images. Automatic segmentation of the optic disc and excavation contours was shown to improve the detection and follow-up of glaucoma progression, which are crucial to ensure timely intervention, preserve vision, and prevent the progression of irreversible VF damage [22,23].

### 2.1. Fundus Photographs and AI

Ophthalmoscopic assessment of the ONH is resource-intensive, making it impractical for large-scale population screening [24]. In this context, DL algorithms have been shown to effectively categorize fundus pictures as glaucomatous or non-glaucomatous by leveraging large datasets. AI typically locates the ONH using its intrinsic characteristics, vascular structural information, or a combination thereof [25] (Table 1).

#### 2.1.1. Segmentation of AI Fundus Images

Recognizing and assessing glaucomatous changes in the optic nerve require a thorough understanding of the characteristics of a normal optic disc.

Current evidence suggests that structural alterations in the ONH and RNFL can occur before VF loss is detectable. For instance, studies have shown that up to 40% of the RNFL can be lost before VF defects are identified through perimetry [32]. Progressive structural changes within the optic nerve are hallmarks of glaucomatous processes. However, similar to other biological variables, the appearance of the optic disc varies significantly among healthy individuals, making it challenging to distinguish between pathological changes.

Manual retinal image inspection remains widely used for diagnosing and monitoring ocular diseases, although it is time-consuming and heavily reliant on physician expertise. Certain conditions require prolonged evaluation over several years for their accurate detection and management.

Optic nerve disorders pose significant diagnostic challenges owing to their variability in appearance, shape, and size in fundus images. The complexity of morphological variations in the optic disc, cup, blood vessels, and PPA complicates automated detection systems, necessitating advanced segmentation techniques and highlighting the need for further algorithm refinement [33].

To enhance efficiency, various medical imaging tools have been developed to detect eye diseases and track disease progression. Some tools compare sequential retinal images to identify changes; however, challenges such as image deformations, texture-less regions, and uneven illumination can hinder precise anatomical alignment [33].

These advancements in computer vision and artificial intelligence have significantly improved glaucoma diagnosis, necessitating automated solutions due to the increasing prevalence of the disease and the subjective nature of current diagnostic methods. Accurate segmentation of the optic cup and optic disc in retinal fundus imaging is essential for glaucoma assessment but remains challenging due to indistinct optic cup boundaries and variations in image properties. Efforts to develop automated diagnostic systems aim to enhance the accuracy and efficiency of glaucoma detection. Precise optic cup and optic disc segmentation provide valuable clinical insights and support the validation of glaucoma screening effectiveness [26].

For the correct assessment of retinal fundus images, which present substantial interindividual variability, Abidin et al. proposed the Efficient Feature Preservation Segmentation Network (EFPS-Net), designed to improve the accuracy of optic cup and optic disc segmentation. EFPS-Net incorporates an atrous residual block in the encoder to enhance feature extraction while maintaining computational efficiency and a recurrent residual block in the decoder to prevent information loss. Additionally, intrinsic feature fusion and extrinsic feature aggregation improved the model’s learning capability and feature retention. In this research, the authors utilized five open access datasets, namely DRISHTI-GS, retinal image database for optic nerve evaluation version 3 (RIM-ONE V3), digital retinal images for the optic nerve segmentation database (DRIONS-DB), the retinal fundus glaucoma challenge (REFUGE), and glaucoma grading from multi-modality images (GAMMA), to conduct experiments. With only 2.6 million parameters, EFPS-Net demonstrates high segmentation accuracy and outperforms state-of-the-art models across five publicly available retinal fundus image datasets. These findings underscore EFPS-Net’s potential in advancing glaucoma diagnosis and treatment, providing healthcare professionals with a powerful AI-driven tool for improved clinical decision-making [26].

Using the U-Net model, Girard et al. introduced a novel glaucoma score, the Atlas Glaucoma Score (AGS), based on a statistical atlas framework, to quantify optic disc deformations caused by glaucoma. This score characterizes the whole deformation of the three anatomical structures inside the OD region: the disc, cup, and vessels. This deformation is obtained by segmenting an OD to be analyzed and then registering it onto the average model of the atlas. It is further expressed as a concise vector corresponding to the projection onto the principal modes of the atlas. The score is finally defined as a linear combination of this vector’s components, with the coefficients learned by a Linear Discriminant Analysis (LDA) classifier using glaucoma and healthy training samples. This score is based on a representation of the local deformations of the OD region, including the vessels, and is less sensitive to the inter-patient variability of the OD shape than the cup-to-disc ratio (CDR). AGS outperformed the traditional CDR in identifying glaucoma, achieving an area under the curve (AUC) of 98.2% across three datasets (i.e., INTERNAL, RIM-ONE, and ORIGA650; n = 13,098) compared to 91.4% for CDR. By capturing the complex optic disc changes that occur before VF loss, AGS shows promise for early glaucoma detection [27] (Table 1).

#### 2.1.2. AI and Optic Disc Classification

The evaluation of the optic disc and peripapillary RNFL (pRNFL) is crucial for identifying glaucoma. While dilated clinical fundus examination is recommended as it provides a stereoscopic view of the optic disc, monoscopic optic disc photographs also offer notable benefits, such as ease of use, cost-effectiveness, and comparable diagnostic accuracy. However, the assessment of optic disc features is labour-intensive and relies heavily on interpretation by skilled specialists.

Li et al. assessed the performance of a DL algorithm for the automated detection of referable glaucomatous optic neuropathy (GON) in a dataset of 48,116 monoscopic fundus photographs. The authors adopted the Inception-v3 architecture, which is a CNN composed of 11 inception modules. The optic disc was analyzed and graded using the model and classified into unlikely, suspect, and certain GON. The DL system achieved an AUC of 0.98, with a sensitivity of 95.6% and specificity of 92.0% in the validation set, indicating its high accuracy in identifying referable GON. False negatives were generally due to high myopia, while false positives were primarily associated with physiological cupping and pathologic myopia [28].

Indeed, anatomical variations in the appearance of the ONH adversely affect DL-based optic disc classification systems. Nam et al. confirmed that ONH tilting negatively impacted the performance of several DL models in labelling pathological changes in the optic disc, including glaucomatous optic disc changes, disc swelling, and pallor. The system employed CNNs with architectures such as VGG16, VGG19, and DenseNet121 to analyze 2507 fundus images and showed a lower classification accuracy for tilted discs compared to non-tilted discs, highlighting the need to account for ONH tilt in future algorithm refinements [29] (Table 1).

#### 2.1.3. AI-Based Prediction of Glaucoma Based on Fundus Photographs

AI algorithms based on fundus photography have shown promise for predicting the development of glaucomatous optic neuropathy. Similarly to the approach used for diabetes complications classification, a similar DL model may be applied to glaucoma detection using fundus images [34]. Just as VGG-19 combined with UNet++ effectively classified abrasion and ischemic diabetic foot sores, a comparable model can be designed to analyze fundus images for glaucoma diagnosis. By leveraging CNNs for feature extraction and UNet++ for precise segmentation of the optic disc and cup, this method can enhance the accuracy of glaucoma detection. Additionally, incorporating data augmentation techniques can improve model robustness against variations in fundus imaging conditions. Evaluating the proposed model against well-known pre-trained classifiers, such as InceptionV3 and MobileNet, can further validate its performance, ensuring a reliable AI-assisted diagnostic tool for glaucoma screening.

In this contest, Thakur et al. assessed the performance of a DL model using 66,721 fundus images from the Ocular Hypertension Treatment Study (OHTS). The DL model (MobileNetV2) was trained on 85% of the images and was validated on the remaining 15%. To identify the regions of the fundus photographs that drive the DL model to assign an image to a glaucoma group or non-glaucoma group, the authors used gradient-weighted class activation maps. Activation maps use the final convolutional layer of a CNN to produce a coarse localization map of the driving regions of the fundus photographs used for diagnosis. While activation maps can be used to validate DL models to verify clinically relevant regions for diagnosis, they can be used to discover potentially novel biomarkers for the disease as well. The model’s AUC for predicting glaucoma onset was 0.77 for predictions made 4–7 years before onset, 0.88 for predictions 1–3 years prior, and 0.95 for post-onset detection, highlighting its increasing accuracy as onset nears [30].

AI-based glaucoma prediction using fundus photographs holds significant promise for early glaucoma detection and management. However, addressing selection bias in the population is crucial to ensure the generalizability and accuracy of these models. Studies have demonstrated that DL models trained predominantly on European and Asian populations may perform poorly when applied to African descent populations [35].

To mitigate such biases, it is essential to incorporate fundus images from various ethnicities, age groups, and ocular conditions to enhance the model’s robustness [35].

In addition, there is a need to ensure consistent imaging protocols across different populations to reduce variability and to test the model on independent datasets from diverse sources to assess its real-world applicability [36] (Table 1).

#### 2.1.4. AI Fundus Picture-Based Tele-Glaucoma

The development and validation of fundus picture-based AI technologies may be particularly helpful for tele-glaucoma, an emergent screening and diagnostic modality that aims to serve remote or under-serviced populations using stereoscopic digital imaging to capture ocular images, which are then electronically transmitted to specialists for evaluation [15]. A systematic review including 45 studies assessed tele-glaucoma as a screening tool for glaucoma, focusing on its diagnostic accuracy, diagnostic odds ratio, and detection rates compared to traditional in-person examinations. The meta-analysis revealed a high accuracy for tele-glaucoma in identifying positive cases, with a higher detection rate than in-person examinations [31].

Nguyen et al. evaluated a DL algorithm within the Los Angeles County Department of Health Services (LAC DHS) teleretinal screening programme to detect referable glaucoma (CDR ≥ 0.6). The DL algorithm, based on the VGG-19 architecture, was trained using patient-level labels generalized to images from both eyes (12,098 fundus images). The algorithm demonstrated high accuracy with comparable or superior performance to that of LAC DHS optometrists and a glaucoma specialist panel. Testing included 1000 images, with metrics like AUC, sensitivity, and specificity used to assess performance [24].

Over the past two decades, several teleophthalmology pilot programmes have been globally implemented. Approximately half of the patients surveyed expressed positive attitudes toward tele-glaucoma programmes, supporting further developments in this field [37] (Table 1).

### 2.2. Visual Field Test and AI

Visual field testing remains the gold standard for the diagnosis and monitoring of glaucoma [38,39]. Standard automated perimetry is preferred in glaucoma management, offering VF assessments through pointwise (PW) sensitivity estimations and global scores (e.g., mean deviation (MD)) and providing tools for computer-assisted interpretation, such as glaucoma progression analysis (GPA) [40].

AI-based approaches have been developed for classifying, staging, and predicting glaucomatous VF loss. These methods include CNNs and variational autoencoders for VF analysis, with some studies also employing recurrent neural networks (RNNs) [41] to incorporate the temporal dimension or archetypal analysis [42] to identify characteristic VF patterns [43] (Table 2).

#### 2.2.1. Convolutional Neural Network-Based Visual Field Test Progression Prediction

A CNN-based approach for predicting VF test progression in patients with glaucoma leverages DL to analyze complex VF data over time. CNNs are particularly suited for image-based data analyses, making them effective for processing VF maps, which represent localized areas of sensitivity in a patient’s VF.

In this regard, using 233 Octopus G1 VF series and the Neural Work Professional II software programme (Neural Ware Ine, Pittsburgh, PA, USA), Brigatti et al. developed a back-propagation neural network model to determine VF progression in patients with glaucoma. The model achieved 73% sensitivity and 88% specificity, closely matching experienced observer evaluations. The output of the back propagation neural network, trained to designate progression as 1 and stability as 0, was a number ranging from 0 to 1. This value represented the “confidence” the neural network has in the assessment of stability or progression. This number is related to probability; the higher the number is, the higher the confidence in the diagnosis of progression [44].

Sample et al. developed a machine learning classifier (MLC) capable of predicting the development of VF abnormalities with a specificity of 96% over a 5.92-year average follow-up period. Based on the analysis of data from 114 ocular hypertensive eyes that had normal VF in baseline examination, the MCL predicted the progression of VF damage in 32% of OHT eyes 3.92 years earlier than conventional methods. Several ML classifiers were compared with the results obtained with SAGE to assess their ability to classify the fields of ocular hypertensive eyes to determine which fields would be classified as abnormal. SAGE is a type of classifier that uses statistically determined cutoffs to distinguish between classes. The ML classifiers were chosen based on the results of a previous study in which we trained a set of classifiers to categorize SAP VF as normal or abnormal [45].

Overall, CNNs appear to enhance VF progression prediction by providing a more detailed analysis of sensitivity patterns and a valuable decision-support tool in managing glaucoma progression. The earlier detection of vision loss by ML classifiers and their use in clinical trials to conduct a quantifiable and comparable evaluation of the data across sites could be very important in the accurate assessment of these new therapies. The ML classifiers detected VF abnormality, on average, 4 years before traditional SAGE classification. In theory, their use could significantly shorten the time of clinical trials assessing small changes in SAP VF over time. The use of appropriate ML classifiers may be even more important in studies in which other methods are used to measure visual function or optic nerve structure (Table 2).

#### 2.2.2. Variational Autoencoder-Based Prediction of Visual Field Test Progression

A variational autoencoder (VAE)-based model for predicting VF test progression in glaucoma patients is an unsupervised DL technique that excels in learning and represents the latent structure of complex VF data. VAEs are generative models that are particularly suited for capturing subtle and progressive changes in VF sensitivity associated with glaucoma.

Berchuck et al. developed a DL algorithm aimed at enhancing the estimation of progression rates and predicting future patterns of VF loss in glaucoma. A generalized VAE was trained to generate a low-dimensional representation of SAP VF, using 29,161 fields from 3832 patients. The model was trained on 90% of the dataset with patient-level randomization, while the remaining 10% was reserved for validation. Using this validation set, the VAE estimated rates of progression and future VF patterns, comparing its performance to traditional SAP mean deviation (MD) rates and point-wise (PW) regression predictions. The VAE-derived longitudinal rate of change identified a significantly higher proportion of progressing eyes than MD at both two years (25% vs. 9%) and four years (35% vs. 15%) from baseline. Additionally, the VAE outperformed PW regression in predicting future VF outcomes, achieving a significantly lower mean absolute error when forecasting the fourth, sixth, and eighth visits based on the first three. These findings demonstrate that a deep VAE model can effectively assess both progression rates and trajectories in glaucoma. Furthermore, as a generative model, the VAE offers the unique advantage of predicting future VF damage patterns, making it a promising tool for glaucoma management [47].

Predicting VF progression in glaucoma remains challenging, and many existing studies lack the integration of AI techniques, which limits their predictive power. Traditional methods for predicting progression often rely on clinical judgement and manual assessment, which can be subjective and prone to error.

AI-based models have the potential to enhance the accuracy of progression predictions by identifying subtle, early changes in VF data that may be missed by human observers. VAE models offer a probabilistic, unsupervised approach to predict VF progression, supporting clinicians in early detection and defining personalized management strategies. For instance, a recent study demonstrated that a DL framework can predict VF early in the disease course, outperforming traditional methods.

However, it is crucial to account for the treatment status in these predictions. Glaucoma treatment can significantly affect the rate of disease progression, and without controlling for treatment variables, predictions may not be meaningful or applicable to real-world scenarios [42] (Table 2).

#### 2.2.3. Recurrent Neural Network-Based Prediction of Visual Field Test Progression

RNNs are a type of neural network model well suited for analyzing time-dependent data because of their ability to capture temporal dynamics through cyclic connections. Unlike feedforward networks, RNNs process data sequentially and maintain memory of previous inputs, allowing them to incorporate information over extended periods. Although traditionally challenging to train and requiring substantial computational resources owing to their numerous parameters, recent advancements in architecture design, optimization methods, and parallel processing have made large-scale RNN training more feasible [49]. This makes RNNs particularly well suited for tasks where context and sequence order are important, such as time-series prediction.

Interestingly, the RNN’s predictive performance reported by Park et al. surpassed that of ordinary linear regression (OLR), particularly in regions prone to glaucomatous damage. Based on a training set of 1408 eyes and a test set of 281 eyes, both the RNN and OLR showed a strong negative correlation with VF MD (Spearman’s rho = −0.734 vs. −0.618). In linear regression analysis, the r^2^ value was 0.380 vs. 0.215 (RNN vs. OLR). The RNN model predicted the future VF significantly better than a conventional pointwise linear regression method simulating the VF outcome printout. This RNN model was also more robust to reductions in the reliability of VF input data.

Thus, the RNN presents a high level of accuracy and reliability in forecasting VF deterioration, making it a valuable tool in clinical decision-making for glaucoma treatment [41] (Table 2).

#### 2.2.4. Archetypal Analysis-Based Prediction of Visual Field Test Progression

Archetypal analysis is a statistical and computational method used to decompose a dataset into a mixture of “archetypes”. Essentially, the archetypal analysis identifies the most representative, extreme patterns within the data, and each observation is expressed as a convex combination of these archetypes [50,51].

In a clinical validation cohort of 397 eyes, of which 27.5% showed confirmed progression, Wang et al. demonstrated significantly higher agreement (kappa of 0.51) and accuracy (0.77) of the archetype method than the Advanced Glaucoma Intervention Study (0.06 and 0.52), Collaborative Initial Glaucoma Treatment Study (0.24 and 0.59), MD slope (0.21 and 0.59), and permutation of pointwise linear regression (0.26, 0.60), with *p* < 0.001 for all comparisons. Compared with existing approaches, this method not only provides progression status but also quantifies the progressed patterns. By decomposing the VF series into 16 archetypes, progression was detected by regressing the archetype coefficients over time [42].

This suggests that quantified information on VF pattern changes over time, and the progression likelihood can be incorporated into the decision-making process of clinicians to yield a more accurate clinical diagnosis of glaucoma progression.

#### 2.2.5. Other ML-Based Methods of Visual Field Test Progression Prediction

Several ML-based methods have been proposed for predicting VF progression in glaucoma. Yousefi et al. developed an unsupervised ML approach, encompassing a mixture of unsupervised Gaussian mixture models with expectation maximization and linear regression to detect longitudinal glaucoma progression from the VF data of 1421 subjects. This approach was tested in a 9-year longitudinal glaucoma cohort and was shown to consistently detect progressing eyes earlier than other methods. A total of 3.5 years of VF data, instead of 5.2 years with traditional methods, were needed to detect progression. Moreover, the model was able to detect more eyes slowly progressing than other methods, providing a prediction printout [48].

To evaluate the ability of several DL networks to predict future VF progression, Wen et al. extracted data from 32,443 consecutive SAPs collected between 1998 and 2018. The CascadeNet-5 model achieved an overall pointwise mean absolute error of 2.47 dB (95% CI: 2.45 to 2.48 dB), demonstrating a statistically significant improvement compared with the linear models. It successfully predicted future HVFs in glaucomatous eyes up to 5.5 years ahead, showing a correlation of 0.92 between the predicted and actual MD; the average difference between the two only accounted for 0.41 dB. These findings indicate that DL networks can effectively learn spatiotemporal changes in HVFs and can provide a representation of forecasted VF predictions based on a single HVF [46].

The ability to anticipate glaucomatous progression may prevent unnecessary functional loss owing to the current practice of multiple confirmatory tests. Incorporating clinical data such as IOP, medication, and surgical history may enhance the efficiency of AI models in clinical decision-making and might facilitate the identification of personalized treatment regimens [46] (Table 2).

### 2.3. Optical Coherence Tomography and AI

Glaucomatous structural damage has been shown to precede VF loss in most cases. To assess glaucomatous progression, optical coherence tomography provides an objective analysis of structural damage to the pRNFL and the ganglion cell layer, ganglion cell complex, and macular ganglion cell–inner plexiform layer (mGCIPL). Given that glaucoma is a three-dimensional (3D) disease characterized by depth-resolved structural changes, fundus imaging may not achieve the same level of accuracy in detecting the diseased status as OCT can [43,52].

The analysis of OCT-derived parameters is useful in the routine clinical care of glaucomatous eyes. However, the presence of anatomical variations, including large peripapillary atrophy, concomitant retinal or ONH disorders, and high axial myopia, has been shown to correlate with segmentation errors. These errors have been reported to occur in up to 36% of the glaucomatous OCT scans. These limitations have driven the development of DL algorithms to improve the quality of OCT images and to remove artefacts. Initial OCT enhancement techniques were not AI-based and focused on correcting light-attenuation artefacts to improve tissue visibility. Recently, several researchers have developed DL methods to denoise and enhance OCT scans [43] (Table 3).

#### 2.3.1. Digital Stain of OCT Images

Devalla et al. developed a DL approach to digitally stain OCT images of the ONH. The dataset included images from one eye of each of the 100 participants (40 healthy and 60 with glaucoma). All images were enhanced through adaptive compensation, and a custom DL network was trained on these enhanced images to digitally stain the six tissue layers of the ONH. The accuracy of the algorithm was evaluated using metrics such as the dice coefficient (0.84 ± 0.03), sensitivity (0.92 ± 0.03), specificity (0.99 ± 0.00), intersection over union (0.89 ± 0.03), and overall accuracy (0.94 ± 0.02) in comparison with manual segmentation techniques. The study also assessed the effects of compensation, the number of training images, and performance differences between OCT scans of the glaucoma and healthy subjects. The DL algorithm successfully stained neural and connective tissues of the ONH, providing a framework for automatically measuring important structural parameters to support glaucoma management [53] (Table 3).

#### 2.3.2. Spectral Domain OCT-Based Glaucoma AI Detection

Ran et al. developed and validated a residual network DL system using a dataset of 4877 SD-OCT volumes of ONH cubes retrospectively collected for training (60%), testing (20%), and primary validation (20%). External validation was performed using three independent datasets from different institutions, and the volumes were labelled for glaucomatous optic neuropathy based on RNFL thinning and corresponding VF defects. Heatmaps were generated for the qualitative assessment. The 3D image-based DL system demonstrated strong performance in detecting GON in both primary and external validations, with an AUC of 0.89–0.89, sensitivities of 78–90%, specificities of 79–86%, and 80–86% accuracy. Heatmaps were generated for qualitative assessments [54].

Garcia et al. proposed a feature extractor from B-scans of SD-OCT volumes centred on the ONH. The dataset included 176 healthy and 144 glaucomatous SD-OCT images and achieved AUC values above 0.93 on both the primary and external test sets. Remarkably, the combined CNN-Long Short-Term Memory system reached an AUC of 0.88 for predicting glaucoma, surpassing other state-of-the-art methods. Class Activation Maps (CAMs) were also generated to identify key regions in each B-scan that could differentiate between healthy and glaucomatous eyes [55].

The importance of combining two different ML models (DL and linear regression) was also reported by Akter et al., who identified clinical features and regions of interest from ONH SD-OCT B-scans that were highly suggestive of glaucoma. Data from 100 glaucoma patients and 100 control subjects, including structural, functional, demographic, and risk factors, were collected and analyzed, and among them, the four most significant features (i.e., MD, pattern standard deviation, cup-to-disc ratio, and RNFL) were used to train several ML algorithms. Subsequently, a new DL model was trained for glaucoma prediction with the aforementioned clinical and imaging data, assessed using five-fold cross-validation. The Grad-CAM produced a deep red colour inside or on the edges of the cup surfaces, which implied that the ONH cup surface area can differentiate between normal and glaucoma groups using the DL technique. Interestingly, the DL model achieved significantly accurate diagnostic performance, with an AUC of 0.98 and an accuracy of 97% on the validation data and 96% on the test data, outperforming previous studies on automated glaucoma detection and supporting its possible clinical use [56] (Table 3).

#### 2.3.3. Anterior Segment OCT-Based AI Algorithms for the Detection of Open vs. Closed Iridocorneal Angle

Anterior segment OCT (AS-OCT) is a useful diagnostic tool for glaucoma diagnosis and subtype classification (i.e., open vs. narrow-angle glaucoma). The AS-OCT imaging technique enables precise quantitative measurements of anterior segment structures, including the iridocorneal angle and anterior chamber depth [61]. This technique can be useful in cases where any condition within the angle-closure spectrum is suspected or during the initial screening of glaucomatous eyes [62].

Xu et al. developed an image-processing and ML-based framework for identifying glaucoma types using OCT images [57]. The proposed method has three significant advantages over existing approaches. First, anterior chamber angle localization from the OCT images was fully automated and efficient across different configurations. Second, unlike previous methods that depend on clinical measurements, it can directly classify the angle as open or closed based solely on visual features. Third, it shows that higher-dimensional visual features surpass lower-dimensional clinical features in angle closure classification accuracy. The framework achieved an AUC value of 0.92 and a balanced accuracy of 84.0% at 85% specificity, outperforming existing methods based on clinical features. Based on a clinical dataset comprising 2048 images, the proposed method required only 0.26 s per image [57].

Niwas et al. investigated the impact of redundant features in diagnosing angle-closure glaucoma mechanisms using AS-OCT images on a dataset of 84 features across 156 samples divided into five classes. An AdaBoost ML classifier was applied to categorize the ACG into five classes: iris roll, lens, pupil block, plateau iris, and no mechanism. The results demonstrate that redundant features selected by the L-score method improved diagnostic accuracy for ACG over the minimum redundancy maximum relevance method [58] (Table 3).

#### 2.3.4. OCT Angiography-Based AI Algorithms for the Detection of Glaucoma

Because the retina is a highly metabolically active tissue, deficiencies in blood flow severely damage energy metabolism and contribute to oxidative damage. Vascular dysfunction, and thus, alterations in autoregulation in response to perfusion pressure changes, may cause an unstable blood supply and, as a result, mild but repeated ischemic injury to the optic nerve. Insufficient blood flow to the retinal structures may be followed by hypoxia, cellular nutrient deficiency, and inefficient waste removal, leading to cellular apoptosis. Alterations in autoregulation in glaucoma appear to involve vascular endothelial dysfunction. Retinal ischemia is a process that involves numerous disorders, including glaucoma [63].

Optical Coherence Tomography Angiography (OCT-A) is an advanced imaging technique that allows the detection of vascular-related diseases affecting the retina and optic nerve. Several clinical studies have shown that OCT-A-based measurements of vessel density and flow index decrease in eyes with glaucoma, particularly in the optic disc, peripapillary retina, and posterior pole. Furthermore, vessel density measured using OCT-A is closely correlated with the severity of VF loss in patients with glaucoma [64].

Given the strong association between glaucoma-related conditions and the vasculature of the retina, OCT-A represents a promising tool for diagnosing and monitoring glaucoma and for advancing our understanding of the disease’s underlying mechanisms [65,66]. However, OCT-A scans can be affected by projection and motion artefacts. To address this issue, AI-based techniques have been developed to remove projection artefacts and improve the visualization of retinal capillaries. In addition, AI can segment retinal vessels, calculate the extent of avascular areas, and create maps of arteries and veins in OCT-A images [65].

In a clinical study, Park et al. evaluated the relationship between macular vessel density and mGCIPL thickness and compared their diagnostic performances. Furthermore, they developed a new combined parameter using an ANN to enhance glaucoma diagnosis. A total of 173 participants were included (100 for testing and 73 for neural network training). The test group included 32 healthy subjects, 33 with early glaucoma and 35 with advanced glaucoma. mGCIPL thickness and vessel density were measured using Spectralis OCT and Topcon swept-source OCT, respectively. Various regression models were used to examine the relationships between macular vessel density and mGCIPL thickness, and a multilayer neural network with one hidden layer was employed to create a single combined diagnostic parameter (i.e., both mGCIPL and OCT-A). A correlation analysis revealed a significant relationship between macular vessel density and mGCIPL (*p* ≤ 0.006). mGCIPL thickness had a much better diagnostic performance for differentiating normal and early glaucoma than macular vessel density. However, incorporating vessel density into mGCIPL thickness using the neural network significantly enhanced the diagnostic performance of the combined parameters compared to mGCIPL thickness alone (*p* ≤ 0.05) [59].

Miguel and Silva recently built a DL software using the OCT-A images, using 90% of them to train its neural network to distinguish the images between glaucoma and non-glaucoma. The remaining 10% was used for validation. The TensorFlow (v.1.15.2) encoder library, followed by several layers of mathematical operations and the RMSProp optimizer and a learning rate of 0.001. Despite the relatively small dataset (i.e., 262 patients, including 40 healthy controls and 222 patients with glaucoma), the results of this pilot study showed promising performance of the AI tool in discriminating glaucoma using OCT-A data, achieving a sensitivity of 99.5%, a specificity of 92.5%, and an AUC of 0.85 [60] (Table 3).

### 2.4. AI Combined Approach in Glaucoma Diagnosis

Predicting the rate of glaucoma progression is crucial for personalized treatment strategies. Structural imaging with optical coherence tomography (OCT) provides a detailed visualization of the glaucomatous optic nerve and retinal damage, while VF tests assess the functional extent of vision loss. However, VF testing is highly variable and patient-dependent, making it challenging to reliably track disease progression.

The structure–function relationship in glaucoma has been extensively studied (Table 4). Previous studies have employed traditional approaches with assumptions about the linearity of this relationship or other structure–function models. However, these models often show a poor correlation between glaucomatous damage and VF defects [67].

A multimodal approach (i.e., fundus photographs and SD-OCT volumes) has been proposed by Miri et al. to leverage the complementary information from fundus photographs and SD-OCT volumes to segment the ONH and cup boundaries [68]. Working on 25 multimodal image pairs from 25 subjects, the authors showed that combining data from fundus photographs and SD-OCT volumes outperformed the unimodal approach (i.e., SD-OCT volumes only) in the automated segmentation of the ONH and optic disc cup [68].

Hussain et al. also developed a multimodal DL model that combines a CNN with a long short-term memory (LSTM) network to predict glaucoma progression. The model was trained on OCT images, VF values, and demographic and clinical data from 86 glaucoma patients over five visits spanning 12 months. This approach predicted VF changes 12 months after the first visit by integrating past multimodal data with synthetic future images generated using a generative adversarial network (GAN).

Patients were categorized into slow progressors (<3 dB VF mean deviation (MD) decline) and fast progressors (>3 dB MD decline). The proposed model achieved an AUC of 0.83 for predicting progression six months earlier. Furthermore, incorporating synthetic future images enhanced early prediction accuracy, allowing the model to anticipate vision loss nine months earlier with an AUC of 0.81, outperforming predictions based on structural data alone (AUC = 0.68) or functional measures alone (AUC = 0.72) [69].

Another approach was attempted by Thompson et al., who trained a DL algorithm to quantify glaucomatous neuroretinal damage on fundus photographs using the minimum rim width relative to BMO (BMO-MRW) from SD-OCT [70]. All optic disc stereophotographs were first preprocessed to derive data for the DL algorithm. The study involved 9282 pairs of optic disc photographs and SD-OCT ONH scans from 927 eyes of 490 subjects. The data were split into validation/training (80%) and test sets (20%). The authors used the residual deep neural network (ResNet34) architecture that had been previously trained on the ImageNet dataset. ResNet is a deep residual network that allows relatively rapid training of very deep CNNs. DL CNN was trained to predict SD-OCT BMO-MRW global and sector values based on optic disc photographs [70]. The DL predictions of the global BMO-MRW from the test set were highly correlated with the observed SD-OCT values (*p* < 0.001) with a mean absolute error of 27.8 μm. The AUCs for distinguishing glaucomatous eyes from healthy eyes using DL predictions and actual SD-OCT global BMO-MRW measurements were 0.945 and 0.933, respectively (*p* = 0.587). Owing to its high accuracy in detecting glaucoma, this software has the potential to eliminate the need for manual segmentation and grading of disc imaging [70].

Xiong et al. developed and validated a multimodal AI algorithm, FusionNet, using pattern deviation probability plots from VF reports and circumpapillary OCT scans to detect GON. DL algorithms, such as FusionNet, learn the relationship between VF and OCT data by identifying informative features during training. The algorithm mimics glaucoma specialist expertise by sharing feature weights between VFNet (i.e., using VF data) and OCTNet (i.e., using OCT data) [71]. FusionNet is based on the bimodal input of the VF and OCT paired data. VF data were collected using a Humphrey Field Analyzer (HFA). OCT images were collected using three types of devices (DRI-OCT, Cirrus OCT, and Spectralis). The 2463 pairs of VF and OCT images were divided into four datasets: 1567 for training (HFA and DRI-OCT), 441 for primary validation (HFA and DRI-OCT), 255 for the internal test (HFA and Cirrus OCT), and 200 for the external test (HFA and Spectralis) [71]. The diagnostic performance of FusionNet was compared to that of VFNet and OCTNet, which showed superior performance. FusionNet was nearly comparable to the glaucoma specialists in the internal and external test sets, evidence indicating the clinical value of multimodal ML models in the automatic detection of GON [71].

### 2.5. AI and Medical Advanced Imaging in Glaucoma

AI has begun to play a crucial role in radiology as well, a discipline heavily dependent on imaging. For instance, magnetic resonance imaging (MRI) can be enhanced virtually using AI models. Computer-assisted diagnosis and detection can support radiologists by performing various tasks, including quantification, segmentation, and preliminary pattern recognition of images [72,73].

AI methods have shown promising results in diagnosing human brain-related diseases [74]. In radiological research focused on the human brain, the primary data consist of MRI. After preprocessing the raw data, images are classified using various ML and DL models, which can mitigate subjective biases inherent to traditional diagnoses by physicians and enable a shift from subjective qualitative analysis to objective quantitative analysis [74].

Although AI has demonstrated significant potential, challenges remain regarding regulatory harmonization, real-world validation, and algorithm generalizability across diverse populations. The integration of AI into ophthalmic practice continues to evolve, with efforts directed toward improving interpretability, patient safety, and clinical trust.

The visual system extends from the eyes to the most posterior segments of the brain, specifically the occipital cortex. Neuro-ophthalmology is an integrative medical discipline focused on the study of disorders affecting the visual pathway [75]. Novel data support the use of MRI in detecting cerebral alterations induced by glaucoma [76,77,78].

The integration of multimodal imaging techniques for glaucoma assessment, as discussed in the paper, provides valuable insights into the potential of DL models for combining structural and functional data. By leveraging methodologies from multi-modal MRI brain tumour segmentation, AI-driven approaches can be adapted to improve glaucoma diagnostics. The ability to analyze and correlate data from different imaging modalities—such as OCT for structural assessment and VF testing for functional evaluation—enhances the accuracy of disease detection and progression monitoring. DL models, particularly CNNs and vision transformers, can effectively process and integrate these diverse datasets, reducing reliance on single-modal assessments. Furthermore, hybrid AI models that combine convolutional and transformer-based architectures offer promising avenues for enhancing feature extraction and segmentation of retinal structures. This multimodal approach could ultimately refine glaucoma diagnosis, aid in early detection, and optimize treatment planning, mirroring advancements seen in brain tumour imaging [79].

In this context, Pang et al. examined the contribution of microstructural and metabolic changes in the brain to glaucoma, and the association with VF loss patterns, employing a multilayer perceptron (MLP) ML algorithm. Advanced diffusion MRI techniques, combined with archetypal analysis, provided unique insights into the potential role of microstructural changes in optimizing compensatory visual function when both eyes are affected by the disease. These data provide complementary insights into VF loss patterns and improve the overall understanding and management of glaucoma [80].

By pursuing this type of clinical study, AI may, in the future, be able to detect early changes in the visual pathway associated with glaucoma and integrate these findings with ophthalmological signs, thereby assisting ophthalmologists in the diagnostic process.

## 3. Ethical Implications

AI applications have greatly impacted healthcare, transforming areas like medical imaging, electronic medical records (EMRs), lab diagnosis, treatments, drug discovery, and personalized medicine. AI also helps in analyzing large amounts of biological data, speeding up healthcare processes, and improving data storage and access. However, the field faces ethical and legal challenges. Although AI enhances treatment and medical practices, its benefits are not equally available, particularly in low-income and developing countries. Ethical concerns such as privacy, data protection, informed consent, and social gaps need to be addressed. Healthcare professionals must ensure AI aligns with the four core medical ethics principles—autonomy, beneficence, nonmaleficence, and justice—before fully integrating it into daily clinical practice workflow [81].

Therefore, new and unique ethical considerations have emerged, necessitating a thorough examination and deliberate resolution [82,83].

Several regulatory documents have already been issued to oversee the application of AI in healthcare. For instance, the General Data Protection Regulation (GDPR) was introduced by the European Union (EU) to enhance privacy protections, influencing similar laws in other countries like the US and Canada. It ensures that all personal data and activities of foreign entities are managed by EU-based data processors or controllers to safeguard individuals’ information. In the US, the Genetic Information Non-discrimination Act (GINA) prevents employers from making decisions based on individuals’ genetic health data.

However, concerns about patient data privacy remain significant to date [81].

Privacy concerns associated with the implementation of AI primarily involve the collection, manipulation, and utilization of data.

In healthcare, data are exposed at two critical points: initially, when entered into electronic medical records, and subsequently, when these records are integrated with AI systems. Data privacy violations can lead to discrimination, loss of insurance or employment, emotional distress, mental health effects, ethical concerns, loss of trust, reluctance to seek care, and harm to vulnerable groups [83].

The introduction of AI also raises concerns regarding the ethical treatment of AI systems. As these technologies advance towards greater autonomy and the ability to make independent decisions, it is essential to address issues related to accountability and the possibility of unintended consequences [82].

AI models used in glaucoma diagnosis and management are susceptible to various biases that can impact their accuracy and generalizability. Selection bias occurs when training data are not representative of the broader population, leading to reduced performance in underrepresented groups. Sampling bias may arise when certain patient groups, such as those with advanced glaucoma, are overrepresented, limiting the model’s ability to detect early-stage disease. Labelling bias is introduced through inconsistencies in clinician-determined ground truth labels, while algorithmic bias can amplify disparities in prediction accuracy across different demographic subgroups. Instrument bias results from variations in imaging devices or diagnostic tools, potentially affecting AI performance when applied to data from different sources. Additionally, confirmation bias can lead AI models to reinforce pre-existing diagnostic patterns, neglecting atypical or emerging presentations of glaucoma. Survivorship bias skews predictions by primarily including patients with long-term follow-up, potentially overlooking individuals with undiagnosed or rapidly progressing disease. Evidence indicates that AI models can amplify human and social biases on a large scale. Data may reflect human decisions or carry the consequences of historical and social inequalities. Additionally, how data are collected and utilized can contribute to biases, with user-generated data potentially reinforcing these issues. Currently, there are no standardized guidelines for reporting and comparing AI models, but future research should address this to support clinicians and researchers.

Addressing these biases is essential to ensuring AI-driven glaucoma diagnostics are fair, reliable, and applicable across diverse patient populations [84].

AI is becoming an integral part of modern digital healthcare systems. As its role in decision-making grows, ensuring that these systems operate ethically and without bias is crucial. There is a pressing need to develop AI systems that are transparent, explainable, and accountable. AI is increasingly being used to improve patient care and surgical outcomes, often surpassing human performance in some areas. As AI continues to evolve, it will either complement or replace current systems, marking the beginning of the healthcare AI era. Not using AI may eventually be seen as unscientific and unethical [84]. Involving clinicians and patients in discussions about medical AI can enhance transparency in areas such as informed consent, human interaction, and fairness. Collaboration between ethicists and clinicians is crucial for addressing the use of AI in diagnosis and treatment, ensuring informed consent, and effectively communicating AI-based diagnoses to patients [85].

It is also mandatory to establish clear responsibility for AI systems’ actions by delineating transparent chains of responsibility for AI and robotic systems. It is important to define the roles and duties of manufacturers, healthcare institutions, and healthcare professionals. Furthermore, incident reporting mechanisms that allow healthcare professionals to promptly report AI system errors or adverse events will be paramount [82].

## 4. Discussion and Future Perspectives

**Figure 2:**
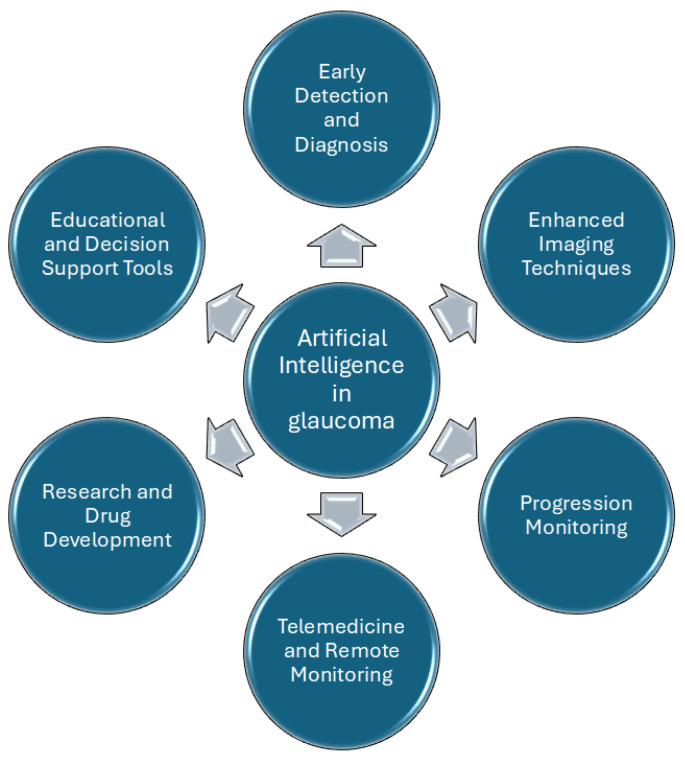
Possible applications of AI in glaucoma.

Integrating AI in healthcare delineates the advent of a new era of medical innovation, promising advancements in diagnostics, streamlined processes, and enhanced patient care. In ophthalmology, AI research efforts are primarily focused on developing tools to enhance disease diagnosis and supporting decision-making processes for ocular conditions. However, most AI systems developed so far are still in the experimental stages, with only few achieving clinical application. Several factors have contributed to the observed delay in applying AI-based tools to routine clinical practice, particularly in glaucoma management [82,86] (Figure 2).

Interestingly, AI-based screening software has been rigorously validated by expert ophthalmologists, especially for diabetic retinopathy (DR). Studies indicate that DL models achieve high sensitivity and specificity (AUC > 0.94) comparable to retinal specialists. AI-driven diagnostic systems demonstrated efficacy in detecting referable DR with sensitivity exceeding 92%. Similarly, AI models analyzing retinal images for glaucoma screening have shown AUC values as high as 0.98, achieving sensitivity levels that rival those of human experts [87].

A review of AI-approved medical devices in ophthalmology revealed a growing number of algorithms achieving regulatory clearance. The USA Food and Drug Administration (FDA) has approved AI-based tools for DR screening, such as IDx-DR (Digital Diagnostics, Ames, IA, USA), AEYE Diagnostic Screening (AEYE-DS; AEYE Health, New York, NY, United States), and EyeArt (Eyenuk Inc., Woodland Hills, CA, USA), which demonstrated high diagnostic accuracy for DR screening [88,89].

In the European Union (EU), the regulatory landscape differs, with a larger number of AI-based ophthalmic devices receiving European certification [89]. AI systems used for ophthalmic diagnostics in the EU include RetmarkerDR (Portugal) and MONA DR (Belgium) for DR diagnosis; OphtAI (France) and RetCAD (Netherlands), which are focused on DR, glaucoma, and age-related macular degeneration (AMD); and DeepDee AI (Netherlands/Belarus) [87].

AI applications extend to optical coherence tomography (OCT) and fundus imaging, enhancing early disease detection. For instance, AI-driven OCT analysis for glaucoma screening achieves an AUC of 0.926, outperforming traditional machine learning techniques. Similarly, DL models used in AMD diagnosis reach a diagnostic accuracy of 91.6% for moderate-to-advanced stages [89].

In the future, the employment of AI in healthcare may transform the field, allowing for significant advancements in diagnosis and treatment, as well as enhancing efficiency and productivity throughout healthcare processes. This can be attributed to the remarkable ability of AI tools to analyze medical images and detect altered patterns in clinical tests, thereby facilitating the early detection of several conditions.

By automating repetitive tasks, healthcare professionals will be eventually able to allocate more of their time and resources towards providing direct patient care, thereby contributing to a higher quality of care for patients and improving patient satisfaction [82].

Current AI models depend on extensive datasets, as insufficient data can substantially degrade their performance. This poses difficulties in AI research for rare eye diseases. Additionally, multimodal and longitudinal cohort studies have stringent requirements for patient data, necessitating prospective data collection, which is both time- and resource-intensive. Consequently, addressing the issue of limited or non-existent data for certain conditions remains a critical challenge [86].

Currently, the most successful AI algorithms in ophthalmology employ fully supervised learning models, which require high-quality data annotation by physicians for specific tasks. This process of medical data labelling is time-consuming and labour-intensive, further complicated by inconsistencies in labelling standards and expertise [86].

Newer weakly unsupervised learning models may reduce reliance on extensive data labelling. However, it is important to note that various image-related factors play a more significant role than technical factors in determining the performance of AI models. Therefore, robust training and testing datasets are essential for effective DL training and application in real-world scenarios [86].

AI-driven models, particularly those utilizing ML and DL techniques, have demonstrated significant potential in optimizing glaucoma management through the analysis of extensive datasets, including clinical records, imaging studies, and patient demographics. The integration of AI into glaucoma care has introduced novel opportunities for enhancing treatment strategies and accurately predicting patient outcomes.

When developing and validating AI models for glaucoma detection and progression monitoring, several confounders can impact the accuracy and generalizability of these algorithms. These confounders include other ocular diseases, prior surgeries, and systemic or topical medications, which may introduce bias or reduce the specificity of AI-based assessments. Potential confounders can significantly impact the performance of AI-based glaucoma detection and monitoring systems. For instance, studies showed that DL algorithms for glaucoma diagnosis based on fundus photographs and OCT tend to have higher false positive rates in myopic populations [29,43]. Similarly, AMD can cause VF defects, which may be misinterpreted as glaucomatous progression [65], and AI models analyzing OCT and fundus images may misinterpret diabetic retinopathy-related changes as glaucomatous damage [25]. Furthermore, AI models trained on high-quality images may struggle with degraded images from patients with cataracts [46]. Finally, the use of medications, such as steroids [59], and surgical intervention [90] may lead to false positive glaucoma classifications. To improve AI robustness and mitigate bias, developers should employ various representative datasets in the AI model’s training stage, including training models on diverse datasets that involve eyes with these confounding conditions [86]. Implementing multimodal AI approaches that integrate fundus photography, OCT, OCT-A, and VF data and adjusting AI models based on surgical history and medication may reduce bias and enhance clinical applicability. By addressing these factors, AI-based systems can become more reliable and generalizable, ultimately improving their clinical utility in real-world glaucoma management.

Developers should continuously monitor AI algorithms for bias and recalibrate them as needed. To do so, collaboration with interdisciplinary teams to evaluate the potential societal impact of AI systems and identify bias in decision-making processes is required [82].

Focusing on glaucoma, the lack of a consensus on a reference standard for diagnosing the disease makes it more difficult to develop diagnostic tests derived from DL algorithms and thwarts the evaluation of new diagnostic tools. While AI already provides an accurate automated diagnosis of eye diseases like diabetic retinopathy using DL algorithms and fundus photography, diagnosing glaucoma remains challenging. This is due to several factors. One of those is the variability in optic disc appearance and the absence of a precise definition of glaucomatous features, raising concerns about the validity of DL models for glaucoma diagnosis from fundus photos. SD-OCT provides objective assessments of neural loss, and the combination of structural data from OCT with VF abnormalities detected by SAP has been shown to enhance diagnostic specificity. However, no shared and objective definition combining SD-OCT and SAP exists for evaluating AI diagnostic algorithms for GON [91]. There have been some attempts in the literature to establish objective criteria for defining GON in open-angle glaucoma. Lyer et al. published a cross-sectional study where data from 2580 eyes (1531 patients) across nine centres on five continents were analyzed to obtain criteria from OCT and VF test results that could define GON objectively [92]. Similarly, a DL algorithm was trained by Mariottoni et al. to detect GON on fundus photos using an objective definition of GON using clearly defined parameters from OCT and SAP. The DL algorithm demonstrated high performance in distinguishing glaucomatous and healthy eyes [91]. However, at present, there is still a lack of consensus on the matter.

Furthermore, glaucoma is a heterogeneous group of progressive optic neuropathies. While the term “glaucoma” is often used generically, multiple distinct subtypes exist, each with unique pathophysiology, clinical presentation, and progression patterns. The most common forms of glaucoma include primary open-angle glaucoma (POAG), which is characterized by normal angle appearance, and major risk factors include the level of intraocular pressure (IOP) and older age; normal tension glaucoma (NTG), a subtype of POAG where optic nerve damage occurs despite there being normal IOP levels, often influenced by vascular and systemic factors; and primary angle-closure glaucoma (PACG), which results from narrow or closed iridocorneal angles, leading to increased IOP due to impaired aqueous humour outflow.

A key question in AI-based glaucoma detection is whether an AI model is subtype-independent or whether certain models are designed for specific types of glaucoma. Some DL (DL) models are trained exclusively on POAG, making their generalizability to NTG or PACG uncertain. For example, AI models based on optic disc appearance (e.g., fundus photography-based models) may struggle with NTG, where ONH damage is more subtle. Anterior segment OCT-based AI models are highly effective for PACG detection but have limited relevance for POAG. VF-based AI models often generalize across different glaucoma types, as functional damage patterns tend to overlap.

Training AI models on a single glaucoma subtype (e.g., POAG) may introduce biases and limit generalizability when applied to broader populations. Conversely, training on multiple glaucoma types can improve AI robustness but may reduce accuracy for specific subtypes due to increased variability in structural and functional features.

To enhance AI effectiveness in glaucoma diagnosis, future research should train AI models using multimodal datasets that include diverse glaucoma subtypes; develop subtype-specific AI models where appropriate (e.g., separate models for POAG vs. PACG); and integrate clinical history, imaging, and functional testing into AI pipelines for a more comprehensive and subtype-aware diagnosis.

This highlights the need for AI validation across diverse glaucoma populations to ensure reliable and clinically meaningful application.

Another complicating factor is the external validation of available algorithms, which is a crucial step in determining the robustness of AI models and their potential translation into clinical practice. Many studies available so far have validated AI models on internal data only, resulting in high diagnostic performance, which may not be replicated on different populations. Regarding diversity, AI-based algorithms should be trained on a racially and ethnically diverse patient population to account for the described variations in several key clinical features, including cup-to-disc ratios (CDRs), intraocular pressure, central corneal thickness, and OCT-derived parameters (e.g., pRNFL and mGCIPL). Evaluating AI models on unseen, large, and heterogeneous datasets is recommended as it represents a step to demonstrate the generalizability, reliability, and efficiency of an unbiased algorithm [93]. On the other hand, overreliance on AI models without critical analysis of the source can amplify the already-discussed biases [94].

Moreover, to ensure fairness in AI-based decision-making, AI algorithms should be developed with a built-in transparency feature that allows healthcare providers to understand the rationale behind recommendations. Implementing explainable AI techniques that provide clear, comprehensible explanations for AI-driven decisions will enhance trust and accountability by stakeholders [82].

For all of these reasons, the clinical implementation and integration of AI are still in their early stages, with ophthalmologists continuing to play a leading role in diagnosing ocular diseases and providing patient care.

The best AI technologies for detecting glaucoma leverage DL and ML models to analyze diagnostic imaging and VF tests. Based on the data provided, some of the most advanced AI techniques perform better in different fields. CNNs detect glaucomatous damage with high accuracy when used for analyzing fundus photographs and OCT scans. VAE unsupervised learning models showed the ability to predict VF test progression with better sensitivity than traditional methods. RNNs were among the most effective for predicting VF deterioration over time by incorporating sequential patient data. FusionNet showed the ability to improve glaucoma detection accuracy combining VF test data and OCT scans. Finally, ANNs gave the best results in enhancing glaucoma diagnosis by analyzing vascular changes in OCT-A scans.

Each of these technologies has been trained on large datasets to improve early glaucoma detection, progression monitoring, and automated diagnosis. Overall, among clinically validated AI systems, some models like Inception-v3, VGG-19, U-Net, and MobileNetV2 have shown promising results in glaucoma classification.

A novel hybrid ensemble approach, similar to those designed for cardiovascular risk prediction [95], may be a starting point to enhance predictive accuracy by aggregating multiple models to reduce variance, bias, and generalization errors in glaucoma. Future research should focus on optimizing model interpretability, reducing data bias, and ensuring clinical validation for AI-assisted glaucoma screening.

## 5. Conclusions

Recent advancements in AI technology for analyzing fundus imaging, OCT, and VF data have demonstrated strong potential in tasks such as classification, segmentation, and glaucoma prediction [96]. These technologies can enhance the accuracy of glaucoma diagnoses by classifying ONH images and aiding in screening for glaucomatous optic neuropathy. Additionally, AI-based glaucoma diagnostic systems could provide significant economic benefits by automating routine tasks, improving diagnostic accuracy, and enhancing access to care, especially in underserved areas. Despite these promising results, challenges persist, including limited dataset size and diversity, class imbalance, the need to optimize models for early detection, and the integration of multimodal data into clinical practice. Currently, ophthalmologists are expected to continue playing a leading role in managing glaucomatous eyes and overseeing the development and validation of AI tools.

## Figures and Tables

**Table 1:** Characteristics of studies aiming to implement fundus photograph-based AI algorithms for the classification, forecasting, and follow-up of glaucomatous optic neuropathy.

Authors	Modality Analyzed	Aims	Methods	Results	Dataset
Nguyen et al. [24]	Fundus Images	Referable glaucoma	VGG-19	DL algorithm approximates or exceeds performance by ophthalmologists (AUC = 0.92) and optometrists (AUC = 0.93)	Training dataset: 12,098 images from 5616 patients Test dataset: 1000 images from 500 patients
Abidin et al. [26]	Fundus Images	Optic disc and cup segmentation	EFPS-Net	Proposed model exhibits superior computational performance with only 2.63 million parameters	
Girard et al. [27]	Fundus Images	Optic disc and cup segmentation	U-Net model	AGS performs better than CDR (AUC= 98.2%)	Datasets: INTERNAL (n = 350), RIM-ONE (n = 159), aRIGA650 (n = 650)
Li et al. [28]	Fundus Images	Referable GON	Inception-v3	DL system achieved AUC of 0.986 with sensitivity of 95.6% and specificity of 92.0%	48,116 fundus photographs
Nam et al. [29]	Fundus Images	OD classification model	CNNs: VGG16, VGG19, DenseNet121	Non-tilted discs’ AUC: 0.98, 0.99, and 0.98 Tilted discs’ AUC: 0.92, 0.92, and 0.93 For VGG16, VGG19, and DenseNet121, respectively	2507 fundus photographs
Thakur et al. [30]	Fundus Images	Glaucoma development prediction	MobileNetV2	Glaucoma development prediction 4 to 7 years before disease onset AUC: 0.77 Model accuracy in predicting glaucoma development 1 to 3 years before disease onset: 0.88 Model accuracy in detecting glaucoma after onset: 0.95	66,721 fundus photographs
Thomas et al. [31]	Meta-analysis	Tele-glaucoma service	Estimates of diagnostic accuracy, odds ratio, and relative percentage of glaucoma cases detected	Tele-glaucoma can accurately discriminate glaucoma with sensitivity of 83.2% and specificity of 79%	45 studies

AGS: atlas glaucoma score; AUC: area under the receiver operating curve; CDR: cup-to-disc ratio; DL: deep learning; GON: glaucomatous optic neuropathy; EFPS-Net: efficient feature preservation segmentation network; OD: optic disc.

**Table 2:** Artificial intelligence-based algorithms developed to determine the status, rate, and pattern of glaucomatous visual field progression.

Authors	Modality Analyzed	Aims	Methods	Results	Dataset
Brigatti et al. [44]	Visual Field	Determine VF progression	Back-propagation neural network	Neural network sensitivity was 73%, and specificity was 88%	233 series of Octopus G1 VF
Sample et al. [45]	Visual Field	Predict development of abnormal VF at follow-up in OHT	Classifiers included two types of support vector machines: constrained MoG and mixture of generalized Gaussian models	ML classifiers predicted abnormality 3.92 years earlier than Statpac-like methods	114 VF
Wen et al. [46]	Visual Field	Forecast future 24–2 HVFs	Fully Connected, FullBN-3, FullBN-5, FullBN-7, Residual-3, Residual-5, Residual-7, Cascade-3, Cascade-5	Up to 5.5 years HVFS prediction with correlation of 0.92 between MD of predicted and actual future HVF	32,443 VF
Berchuck et al. [47]	Visual Field	Estimate rates of progression and predict future patterns of VF loss in glaucoma	Variational auto-encoder	VAE detected significantly higher proportion of progression than MD at two (25% vs. 9%) and four (35% vs. 15%) years from baseline	29,161 VF
Park et al. [41]	Visual Field	Predict future VF damage	RNN	RNN and OLR showed strong negative correlation with VF MD (Spearman’s rho = −0.734 vs. −0.618); in linear regression analysis, r^2^ was 0.380 vs. 0.215 (RNN vs. OLR)	Training dataset: 1408 eyes. Test dataset: 281 eyes
Wang et al. [42]	Visual Field	Detect VF progression	Archetype method	Agreement (kappa) and accuracy of archetype method (0.51 and 0.77) significantly (*p* < 0.001 for all) outperformed AGIS (0.06 and 0.52), CIGTS (0.24 and 0.59), MD slope (0.21 and 0.59), and PoPLR (0.26 and 0.60)	Development cohort: 11,817 eyes Clinical test dataset: 397 eyes
Yousefi et al. [48]	Visual Field	Detect glaucoma progression	Unsupervised Gaussian mixture model with expectation maximization	ML analysis detects progressing eyes earlier (3.5 years) than other methods consistently; ML detects more slowly progressing eyes than other methods (5.1 years)	VF of 1421 subjects.

AGIS: Advanced Glaucoma Intervention Study; CIGTS: Collaborative Initial Glaucoma Treatment Study; HVFs: Humphrey visual fields; MD: mean deviation; ML: machine learning; MoG: mixture of Gaussian; OHT: ocular hypertensive; OLR: ordinary linear regression; PoPLR: permutation of pointwise linear regression; RNN: recurrent neural network; VAE: variational auto-encoder; VF: visual field.

**Table 3:** OCT-based artificial intelligence models discussed in this review.

Authors	Modality Analyzed	Aims	Methods	Results	Dataset
Devalla et al. [53]	SD-OCT	Digitally stain OCT images of ONH	Custom DL	Dice coefficient (0.84), sensitivity (92%), specificity (99%), intersection over union (0.89 ± 0.03), and accuracy (94%)	100 eyes
Ran et al. [54]	SD-OCT	Detect GON	Residual network	AUROCs of 0.89–0.89, sensitivities of 78–90%, specificities of 79–86%, and accuracies of 80–86%	4877 SD-OCT volumes of optic disc cube
Garcia et al. [55]	SD-OCT	Glaucoma prediction	LSTM network	In prediction stage: AUC > 0.93 both in primary and external test sets. Combination of CNN and LSTM networks achieves AUC = 0.88	176 healthy and 144 glaucomatous SD-OCT volumes
Akter et al. [56]	SD-OCT	Diagnostic glaucoma assessment	CNN architecture	DL model trained from optimal features: AUC = 0.98 and accuracy of 97% on validation data and 96% on test data DL model used in pilot study: AUC = 0.99 and accuracy of 98.6%	200 subjects, consisting of 100 healthy subjects and 100 subjects with glaucoma
Xu et al. [57]	AS-OCT	Identify glaucoma type	Image processing and machine learning-based framework	Proposed method only requires 0.26 s per image; framework achieves 0.92 AUC value and 84.0% balanced accuracy at 85% specificity	2048 images
Niwas et al. [58]	AS-OCT	Complex disease diagnosis	L-score and MRMR algorithms, AdaBoost	Study found that unsupervised L-score method achieved classification accuracy of 86.6% using 40 features Supervised MRMR method reached accuracy of 79.3% with 40 features and 84.3% with smaller set of 10 features	84 features and 156 samples
Park et al. [59]	OCTA	Diagnostic performance of macular vessel density and GCIPLT	Multilayer neural network	When incorporated into macular GCIPL using artificial neural network, combined parameter showed better performance than macular GCIPL alone	173 subjects
Miguel et al. [60]	OCTA	Assist in glaucoma diagnosis	Custom DL software	AI system successfully discriminated glaucoma from healthy eyes based on OCT-A scans with sensitivity of 99.5%, specificity of 92.5%, and AUC of 85%	262 patients

AS-OCT: anterior segment OCT; AUC: area under the receiver operating characteristic curve; CNN: convolutional neural network; DL: deep learning; GCIPL: ganglion cell to inner plexiform layer; GON: glaucomatous optic neuropathy; L-Score: Laplacian score; LSTM: long short-term memory; MRMR: minimum redundancy maximum relevance; OCT: optical coherence tomography; OCT-A: optical coherence tomography angiography; ONH: optic nerve head; SD-OCT: spectral domain optical coherence tomography.

**Table 4:** Artificial intelligence models using multimodal imaging discussed in the present review.

Authors	Modality Analyzed	Aims	Methods	Results	Dataset
Miri et al. [68]	Multimodal	Optic disc and cup boundary segmentation	Unimodal and two multimodal machine-learning graph-based approaches for automated segmentation of optic disc and cup	Multimodal approaches outperform the unimodal approach in segmenting the optic disc and cup.	25 multimodal image pairs from 25 subjects
Hussain et al. [69]	Multimodal	Predict glaucoma progression	Multimodal DL model that combines CNN with LSTM network	The proposed model achieved an AUC of 0.83 for predicting progression six months earlier.	Model was trained on OCT images, VF values, and demographic and clinical data from 86 glaucoma patients over five visits spanning 12 months
Thompson et al. [70]	Multimodal	Quantify glaucomatous neuroretinal damage	DL CNN	AUCs for discriminating glaucomatous from healthy eyes with the DL predictions and actual SD-OCT global BMO-MRW measurements were 0.94 and 0.93, respectively (*p* = 0.587).	Total of 9282 pairs of optic disc photographs and SDOCT optic nerve head scans
Xiong et al. [71]	Multimodal	Detect GON	FusionNet based on bimodal input of VF and OCT paired data were developed to detect GON	FusionNet achieved an AUC of 0.95.	2463 pairs of VF and OCT images from 1083 patients

CNN: convolutional neural network; GON: glaucomatous optic neuropathy; LSTM: long short-term memory; OCT: optical coherence tomography; SD-OCT: spectral domain optical coherence tomography; VF: visual field.
